# Antimicrobial susceptibility testing of *Staphylococcus aureus* isolates from patients at a tertiary hospital in Tehran, Iran, 2018–2019

**DOI:** 10.1186/s40001-022-00778-w

**Published:** 2022-08-17

**Authors:** Mohammad Qodrati, SeyedAhmad SeyedAlinaghi, Seyed Ali Dehghan Manshadi, Alireza Abdollahi, Omid Dadras

**Affiliations:** 1 School of Medicine, Tehran University of Medical Sciences, Tehran, Iran; 2 Iranian Research Center for HIV/AIDS, Iranian Institute for Reduction of High-Risk Behaviors, Tehran University of Medical Sciences; Imam Khomeini Hospital Complex, Keshavarz Blvd., Tehran, 1419733141 Iran; 3 Department of Infectious Diseases and Tropical Medicine, Imam Khomeini Hospital Complex, Tehran University of Medical University, Tehran, Iran; 4 Division of Pathology, Imam Khomeini Hospital Complex, Tehran University of Medical Sciences Tehran, Tehran, Iran; 5 Section Global Health and Rehabilitation, Western Norway University of Applied Sciences, Bergen, Norway

**Keywords:** *Staphylococcus aureus*, MRSA, Antimicrobial resistance, Antimicrobial susceptibility testing, Empiric therapy, Iran

## Abstract

**Background:**

*Staphylococcus aureus*, a human skin and mucous membranes colonizer, could opportunistically cause a variety of infectious diseases. Frequently, it is resistant to methicillin (MRSA), and often, co-resistant to many clinically available antibiotics. MRSA is a major burden for healthcare systems and communities all over the world, especially in developing countries. We addressed the issue that more than a decade had passed since the last report about cumulative antibiogram for *S. aureus* from our center, whereas The Clinical and Laboratory Standards Institute (CLSI) recommends to analyze and report it on an annual basis in order to guide clinicians to select the best initial empiric antimicrobial therapy.

**Methods:**

In a cross-sectional retrospective design, data of culture-proven *S. aureus* from clinical specimens of hospitalized patients at Imam Khomeini Hospital Complex, Tehran, Iran, were collected from September 2018 to September 2019. Antimicrobial susceptibility testing (AST) had been performed using either Kirby–Bauer disk diffusion or VITEK 2 automated system which is based on minimum inhibitory concentration (MIC). The Chi-squared test was used considering the critical *p*-value to be ≤ .05.

**Results:**

Among 576 unique isolates, the overall prevalence of MRSA was 37.5%. Patients admitted to the infectious diseases ward and ICUs have a greater chance to have such an isolate. Methicillin resistance was predictive of resistance to most antibiotics: erythromycin (90.9%), clindamycin (85.4% including inducible resistance), gentamicin, cipro-/levo-/moxi-floxacin, trimethoprim–sulfamethoxazole (58.3%), tetracycline, and rifampin. Resistance rate of zero was observed for daptomycin, linezolid, tigecycline, and (roughly) vancomycin. The prevalence of multiple-drug resistant (MDR) isolates was 48.5%.

**Conclusions:**

Although in this study, the prevalence of MRSA was lower than the previous ones from the same hospital, it is still far from the desired rates. Besides, resistance to clindamycin and trimethoprim–sulfamethoxazole were remarkable. So far, vancomycin is the best choice for empiric treatment of MRSA, with linezolid as the second choice. It is advised to avoid prescribing the newer antibacterial agents as long as the older ones are effective to prevent the emergence of MDR species.

## Background

*Staphylococcus aureus* is one of the most common colonizers and a cause of different infections [1]. Ogston’s coccus [2], officially named by Rosenbach [3], has a strong capacity to develop resistance against virtually all antibiotic classes. *S. aureus* isolates reportedly became resistant against penicillin within one to two years, methicillin within less than a year [4], and vancomycin about 40 years [5] since their clinical introduction. Because the mechanism of resistance alters the target of the antibiotic, resistance against an agent in vitro usually indicates clinical resistance against all the other agents in the same class, even though one of them may appear to be effective in vitro [6]. Simultaneously, multiple-drug resistance (MDR) against different classes may coexist through different mechanisms as well.

Methicillin resistance in *S. aureus* (MRSA) may be considered per se as another definition for multiple-drug resistance [7]. It correlates with several epidemiologic features [8] and could signalize increased resistance against other agents (for example, clindamycin) [9]. Antimicrobial treatment naturally exerts selection pressure of MRSA and other resistant isolates but, commonly in developing countries, the inappropriate use of antibiotics for community infections may be another cause for increased resistance. Meanwhile, the higher prevalence of MRSA in developed countries may suggest the injudicious use of prescription or over-the-counter antimicrobial medicines.

In the current era, as new potent antibiotics have been merely produced and clinically approved, it is becoming more important to use anti-staphylococcal agents judiciously; try the older agents with a narrow/targeted spectrum at the first lines by an appropriate dose and duration; hesitate prescribing antibiotics where no evidence-proven indication exists; and wait for the antibiogram results if the situation permits. Also, monotherapy of *S. aureus* infections with rifampin (RIF) or fluoroquinolones (FQ) should be avoided because of the rapid emergence of resistant mutants [10]. The “seesaw effect” is another hope, which demonstrates improved beta-lactam activity when glyco- and/or lipopeptides susceptibility decreases [11].

The Clinical and Laboratory Standards Institute (CLSI) M39 recommends analyzing and presenting cumulative antibiogram reports at least annually to be mostly used in guiding initial empiric antimicrobial therapy decisions in patients for whom microbiological test data to target treatment do not yet exist [12]. We addressed the issue that more than a decade had passed since the last such report for *S. aureus* from our center.

## Methods

### Study design and participants

This cross-sectional retrospective study was conducted at Imam Khomeini Hospital Complex, a tertiary referral care center and university hospital in central Tehran, Iran. Clinical samples of various specimen types were collected from all hospitalized patients in different wards from September 2018 to September 2019. General, neonatal, cardiac, and other specialties’ intensive care units (ICUs) involved in the study, as well as emergency department, surgical, neurosurgical, orthopedics, and otorhinolaryngology wards and operation rooms; internal medicine, dermatology, neurology, infectious diseases, obstetrics and gynecology, and pediatric wards. Specimen types were considered as follows: blood; wound secretions; respiratory secretion and sputum; abscess, tissue, bone, and intra-articular fluid; urine; pleural, peritoneal, and pericardial fluids; catheters and devices; and others. Data of *S. aureus* isolates were collected from the medical records. Repeat isolates were excluded following the CLSI M39 recommendations on a patient basis; the first isolate per patient in a one-year period was analyzed, irrespective of the body site from which the specimen was obtained or the antimicrobial susceptibility pattern [13]. Isolates with missing data were also excluded.

### Measurement and interpretation

In this study, we used phenotypic methods for identification and antimicrobial susceptibility testing (AST) of *S. aureus* isolates. To this end, each specimen underwent testing with a sequence of identification methods including Gram-stained smears light microscopy, observation of growth pattern and colony morphology on various media (including deoxyribonuclease agar and mannitol salt agar), manual biochemical reactions (catalase and coagulase tests), or the use of BACT/ALERT® (bioMérieux) and VITEK 2® COMPACT (bioMérieux) automated systems whenever the specimen was compatible and the required consumable materials were available.

Dilution methods (including broth microdilution), which can measure the minimum inhibitory concentrations (MIC) of antibiotics, are considered the gold standards for phenotypic AST. Whenever possible, we used the aforementioned automated system which performs this method. On the other hand, we often used Kirby–Bauer disk (BD BBL; Rosco; Mast) diffusion method on Mueller–Hinton agar (Ibresco; Conda) plates for manual AST. It is considered the cheapest and most simple method for susceptibility testing. Isolates evaluated using the latter method were also routinely tested for inducible clindamycin resistance by D-test.

The measured MICs and inhibitory zone diameters were interpreted using CLSI M100 guidelines [14]. Notably, an *S. aureus* isolate was considered resistant to methicillin (MRSA) when oxacillin MIC was ≥ 8 μg/mL or when there was an inhibitory zone diameter of ≤ 21 mm around a 30-μg cefoxitin disk which is acceptable and feasible in place of genetic methods [15].

Resistance against vancomycin was routinely determined similarly although the disk diffusion method is not recommended anymore. The MIC was measured if doubtful results occurred or a request by the responsible physician was placed.

To calculate the overall rate, MDR was defined as non-susceptibility to ≥ 1 agent in ≥ 3 antimicrobial categories. To compare MDR rates between methicillin-sensitive *S. aureus* (MSSA) and MRSA isolates, we omitted the beta-lactams as being an antimicrobial category. In this study, an antibiotic susceptibility (or resistance) pattern indicates the antibiotics to which the isolate is susceptible (or resistant) simultaneously.

### Statistical analysis

Data were gathered and cleaned using Microsoft Office Excel. Different antibiotic susceptibility or resistance patterns and their frequency were calculated by a custom Python script. Finally, data were imported into and analyzed using IBM® SPSS® Statistics, version 26. The Chi-squared test was used to determine the significance of the observed difference between groups, considering the critical *p*-value to be ≤ .05.

## Results

After removing outpatient and repeat isolates, 576 unique *S. aureus* isolates (60.2% of all inpatient isolates) were analyzed (Table 1). The number of antibiotics tested varied from 1 to 17 per isolate; the mode, the mean, and the standard deviation were 7, 7.8, and 3.3, respectively. Overall, the relative prevalence of MRSA was 37.5%.

**Table 1:** Baseline characteristics of received clinical specimens, of which *Staphylococcus aureus* isolated at IKHC, Tehran, Iran, 2018–2019

Variable	*S. aureus* (%)	MRSA (%)	p-value
Ward			
Emergency	207 (35.9)	60 (29.0)	.002
Internal, Dermatology	106 (18.4)	44 (41.5)	
ICU, NICU, CCU	90 (15.6)	47 (52.2)	
Surgical wards, Neurosurgery, Operation rooms	121 (21.0)	40 (33.1)	
Infectious diseases	24 (4.2)	14 (58.3)	
Obstetrics and Gynecology, Pediatric	28 (4.9)	11 (39.3)	
Specimen type			
Blood	298 (51.7)	119 (39.9)	.078
Wound secretions	64 (11.1)	25 (39.1)	
Respiratory secretions and sputum	36 (6.2)	15 (41.7)	
Abscess, tissue, bone, intra-articular fluid	81 (14.1)	21 (26.0)	
Urine	24 (4.2)	13 (54.2)	
Pleural, peritoneal, and pericardial fluids	38 (6.6)	10 (26.3)	
Catheters and devices	13 (2.3)	5 (38.5)	
Others	22 (3.8)	8 (36.4)	

*ICU* intensive care unit, *NICU* neonatal ICU, *CCU* coronary care unit

**Fig. 1:**
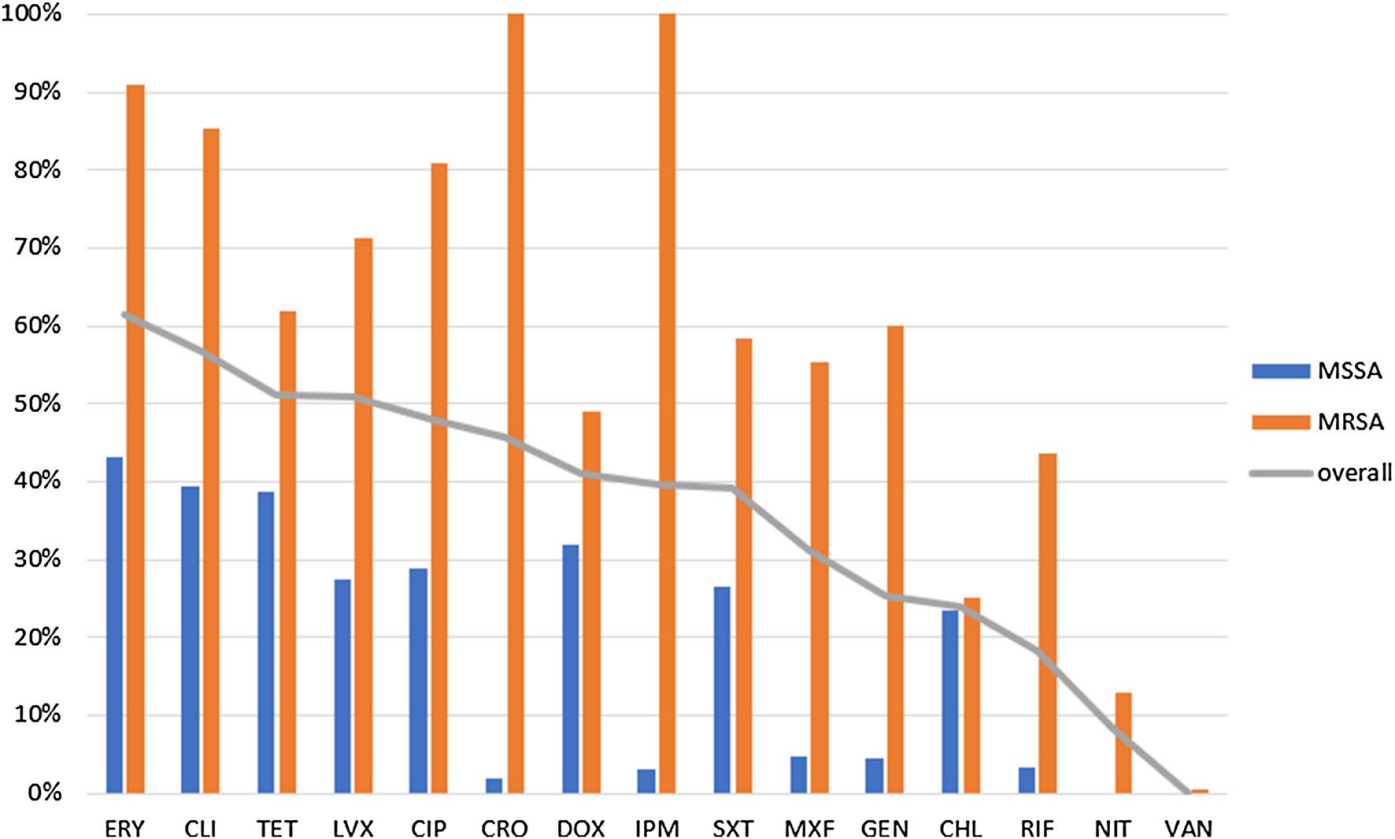
Resistance rates of *Staphylococcus aureus* isolates at IKHC, Tehran, Iran, over one year (2018–2019). Resistance against linezolid, daptomycin, and tigecycline were not seen

The emergency department (35.9%) and blood specimens (51.7%) were the most frequent origins of the *S. aureus* isolates. More than a half of *S. aureus* isolates were MRSA in infectious diseases ward (58.3%) and ICUs (52.2%), while MSSA were the most frequent isolates (71%) that would be obtained from clinical specimens in the emergency department (Fig. 1).

MRSA isolates were resistant against erythromycin (ERY), clindamycin (CLI), ciprofloxacin (CIP), and levofloxacin (LVX) at the rate of > 70%. All resistance rates for MSSA isolates were < 50% against each of the tested antibiotics.

Overall, MDR rate was 48.5% and it was significantly different (*p*-value < .001) between MRSA (65.5%) and MSSA (24.7%) isolates given the omission of beta-lactams from the drug resistance definition.

Reporting the more frequent antibiogram patterns in Table 2, we did not include nitrofurantoin (NIT) as it is mainly used in urinary tract infections. Also, at least 30 isolates were tested against these patterns in accordance with CLSI M39 guidelines.

**Table 2:** The most frequent (%) non-beta-lactam co-resistance patterns of *Staphylococcus aureus* isolates at IKHC, Tehran, Iran, 2018–2019

MSSA	MRSA	Overall
CLI/ERY (33.6)	CLI/ERY (84.6)	CLI/ERY (53.0)
DOX/TET (29.8)	CIP/ERY (77.7)	CIP/LVX (50.9)
CIP/ERY/LVX (27.5)	CIP/CLI (75.5)	CIP/CLI/ERY/LVX (49.5)
CIP/CLI/ERY/LVX/SXT (26.7)	CIP/CLI/ERY (75.3)	CIP/CLI/LVX (49.1)
CIP/CLI/LVX (26.5)	CIP/LVX (71.2)	CIP/ERY (44.9)
CIP/CLI/ERY/LVX (26.1)	CIP/CLI/ERY/LVX (67.8)	CIP/CLI (43.7)
ERY/TET (26.1)	ERY/GEN (60.6)	CIP/CLI/ERY (43.0)
CIP/DOX (26.0)	CIP/GEN (59.2)	CLI/TET (41.2)
CLI/TET (25.5)	CLI/ERY/GEN (59.2)	ERY/TET (40.6)
CIP/CLI/LVX/SXT CIP/ERY/LVX/SXT (25.5)	CIP/ERY/GEN (58.8)	CIP/LVX/TET (40.4)

*MSSA* methicillin-sensitive *Staphylococcus aureus*, *MRSA* methicillin-resistant *S. aureus*, *CIP* ciprofloxacin, *CLI* clindamycin, *DOX* doxycycline, *ERY* erythromycin, *GEN* gentamicin, *LVX* levofloxacin, *SXT* trimethoprim–sulfamethoxazole, *TET* tetracycline

**Fig. 2:**
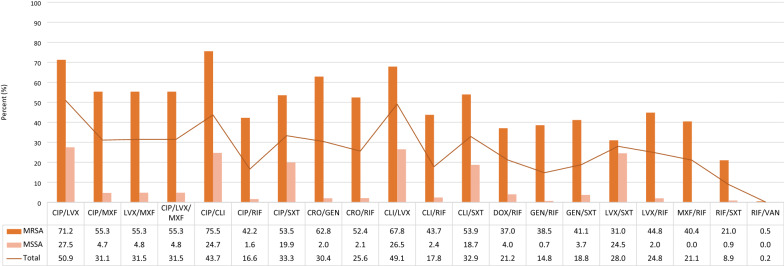
Co-resistance (%) of *Staphylococcus aureus* isolates against some clinically important antimicrobial patterns at IKHC, Tehran, Iran, 2018–2019

Alternatively, Fig. 2 provides the relative frequencies of some clinically important patterns which are mostly required to decide about the treatment regimens.

In addition, the most frequent co-susceptibility rates belonged to MXF/RIF overall (81.0%), and to CHL/RIF for MRSA (67.5%) isolates.

## Discussion

The purpose of this study was to determine cumulative antibiograms (Table 3), as CLSI M39 recommends [12], for *Staphylococcus aureus* isolates in our center to incorporate in antibiotic stewardship programs. After analyzing 576 unique *S. aureus* isolates from clinical specimens, the overall prevalence of MRSA isolates was 37.5%. More than 80% resistance rates against ERY, CLI, CIP, and LVX were seen among MRSA isolates which is alarming as CLI and FQ are of the most empirically prescribed antibiotics by our clinicians. No resistance was found against linezolid (LZD), daptomycin, and tigecycline, and the only in vitro-resistant isolate against vancomycin (VAN) had no clinical importance.

The overall prevalence of MDR isolates was 48.5% in our study, which is almost equal to the rate calculated by Dilnessa and Bitew [9] in Addis Ababa (including beta-lactams; 50.5%), higher than what Wiliamson et al. [16] reported from New Zealand (omitting beta-lactams; 6%), and lesser than what was in the study of Kim et al. [17] with a customized definition (97.7%).

In our study, MRSA prevalence rate was lower than previous reports from the same center by Khalili et al. [18] and Mohraz et al. [19], lower than the overall rate in Iran (52.7%) and almost equal to the least value reported from Tehran Province through a review and meta-analysis by Askari et al. [20]. It was much lower than the 96.1% rate which Yadegarynia et al. [21] found in another hospital in Tehran. Doing a comparison of isolates causing invasive infections from 29 European countries in 2018, we would be placed after Romania, Cyprus, and Portugal, in fourth place of the most MRSA-prevalent countries; the overall rate in Europe is 19.3% in the same report [22].

Comparing each ward to the others and also the overall population, MRSA prevalence was observed to be significantly higher in infectious diseases ward and ICUs, while it was significantly lower only in the emergency department. There was no statistically significant difference between the MRSA prevalence in the other wards and the overall prevalence. Therefore, *S. aureus* isolates could be presumed MRSA only in the infectious diseases ward and the ICUs. The above results are reasonable; most community-onset infections, which are associated to less resistant organisms, present to the emergency department in comparison to healthcare-associated infections caused by more resistant pathogens in the inpatient wards, which also increase the overall resistance rate.

Mohraz et al. [19] found that the general ICU had the most and, in contrast to our study, the infectious diseases ward has the least MRSA rates. A promising result from our study shows that the prevalence of MRSA in ICUs was 52.2% which is much lower than what Rashidi Nezhad et al. [23] reported from seven hospitals in Tehran, and slightly lower than that had been in this center based on Khalili et al. [18]. Again, we have more than twice the MRSA:MSSA rate that the European Centre for Disease Prevention and Control has reported from healthcare-associated infections in ICUs [24].

MRSA rates were not significantly different between specimen types in our study. This was opposed to what was found by Mohraz et al. [19], Waitayangkoon et al. [8], or Dilnessa and Bitew [9]. The reason may be the multitude of types in our study.

Resistance status against most antibiotics was significantly higher with methicillin resistance; 100% resistance against the other beta-lactams (ceftriaxone, imipenem) was naturally expected. A MRSA isolate would, more probably, be resistant to FQ, CLI, TET, ERY, gentamicin (GEN), RIF, and SXT and no difference from MSSA was seen against CHL, DOX, NIT, and VAN. These findings were in line with other studies [9, 25, 26]. Highest co-resistance was shown against pairs containing a commonly used FQ (i.e., CIP, LVX) plus an adjunctive agent, so it may be representative of their inappropriate usage as monotherapy. The most frequent susceptibility pattern was to the RIF-based regimens, but clinical data have not demonstrated better results than standard therapies without RIF [27].

Resistance to clindamycin in our study, which included inducible clindamycin resistance by our laboratory routines, was 56.8% overall and 85.4% for MRSA isolates. The Infectious Diseases Society of America (IDSA) guidelines recommend [28] treating skin and soft tissue MRSA infections empirically with clindamycin when a low resistance rate (e.g., 10%) is present. Therefore, our results do not support the empiric use of clindamycin in this center.

Considering the limitation of the disk diffusion method to determine vancomycin resistance, it was seen in only one isolate; a MRSA which was simultaneously resistant to all other tested antibiotics (i.e., CIP, CLI, ERY, GEN, RIF, SXT). However, it might not be truly vancomycin-resistant because the clinical infection was resolved with the administration of vancomycin. Other isolates seemed to be sensitive based on the available clinical records. Although high-level vancomycin-resistant *S. aureus* isolates were reported from the same center [29] and the vancomycin-intermediate *S. aureus* prevalence rate is reportedly 0.90% in Iran [30], our results seem to be promising.

LZD is more clinically available and the only oral choice out of the three newer agents with 100% susceptibility rates in our study. Similar rates were observed by others [31, 32], but the emergence of LZD-resistance has already begun and is a progressive trend over time as shown by multiple studies like Baddour et al. [6] with a 4.1% resistance rate. Although these agents are valuable additions to our antimicrobial options, we should limit their use to the patients who truly require them, to postpone the inevitable emergence of antibiotic resistance in the world.

Doing a retrospective record review on sparse data written into paper and electronic records in a large university center of different medical specialties with resource shortage, we did a lot of work to collect, authenticate, and prune as much information as possible. The quality assurance measures were considered in several steps; laboratory works were performed by different technicians using the best equipment available at the time for that specimen type, meeting the needs of the responsible physician. Therefore, each specimen was evaluated through manual or automated methods. Available antimicrobial agents (disc and cards) to test were not the same over the study period, and VAN resistance is not perfectly reliable because of the routine method in our center.

**Table 3:** One-year cumulative antibiogram of unique *Staphylococcus aureus* isolates at IKHC, Tehran, Iran, 2018–2019

Antibiotic	MSSA 360 (62.5%)			MRSA 216 (37.5%)			*p*-value
	S	I	R	S	I	R	
CRO	52	–	1	–	–	43	< .001
CHL	13	–	4	6	–	2	> .9
CIP	239	3	94	38	4	156	< .001
LVX	37	6	8	17	9	33	< .001
MXF	41	–	2	21	8	18	< .001
CLI	213	–	139	31	-	182	< .001
DAP	32	–	–	25	–	–	–
LZD	51	–	–	51	–	–	–
TGC	41	–	–	35	–	–	–
DOX	34	6	10	28	11	16	.20
TET	30	–	19	21	–	34	.019
ERY	184	2	138	18	3	177	< .001
GEN	298	–	14	75	2	111	< .001
IPM	32	–	1	–	–	20	< .001
NIT	17	–	–	27	3	1	.380
RIF	326	–	11	112	–	87	< .001
SXT	184	–	66	70	–	98	< .001
VAN	358	–	–	214	–	1	.375

*MSSA* methicillin-sensitive *Staphylococcus aureus,* *MRSA* methicillin-resistant *S. aureus*, *S* sensitive, *I* intermediate resistance, *R* resistant, *CRO* ceftriaxone, *CHL* chloramphenicol, *CIP* ciprofloxacin, *LVX* levofloxacin, *MXF* moxifloxacin, *CLI* clindamycin, *DAP* daptomycin, *LZD* linezolid, *TGC* tigecycline, *DOX* doxycycline, *TET* tetracycline, *ERY* erythromycin, *GEN* gentamicin, *IPM* imipenem, *NIT* nitrofurantoin, *RIF* rifampin, *SXT* trimethoprim–sulfamethoxazole, *VAN* vancomycin

## Conclusions

Overall, the prevalence of MRSA in this study was lower than the previous ones from the same hospital; but it is still far from the desired rates. Also, resistance to well-known alternative antibiotics such as clindamycin and trimethoprim–sulfamethoxazole appeared to be unacceptably high. It may be more reasonable to empirically start with the first-generation cephalosporins instead of clindamycin when *S. aureus* infection is suspected, and the natural course and response to the treatment should be further considered in escalating the antimicrobial regimen. So far, the injectable-only vancomycin is the gold standard for the treatment of MRSA infections because of the low resistance rate, as well as its availability compared with the newer agents which have higher costs and side effects. Linezolid is the only oral agent that became favored to treat MRSA infections; however, it is better to reserve such agents as the last resort when the vancomycin resistance rate reaches a significant level in the future.

## Data Availability

The datasets analyzed during the current study are available from the corresponding author on reasonable request.

## Abbreviations

MRSA: Methicillin-resistant *Staphylococcus aureus*
MSSA: Methicillin-sensitive *Staphylococcus aureus*
CLSI: The Clinical and Laboratory Standards Institute
AST: Antimicrobial susceptibility testing
ICU: Intensive care unit
IKHC: Imam Khomeini Hospital Complex
MDR: Multiple-drug resistant/resistance
CHL: Chloramphenicol
CIP: Ciprofloxacin
CLI: Clindamycin
CRO: Ceftriaxone
DAP: Daptomycin
DOX: Doxycycline
ERY: Erythromycin
GEN: Gentamicin
IPM: Imipenem
LVX: Levofloxacin
LZD: Linezolid
MXF: Moxifloxacin
NIT: Nitrofurantoin
RIF: Rifampin
SXT: Trimethoprim–sulfamethoxazole
TGC: Tigecycline
TET: Tetracycline
VAN: Vancomycin
